# Resilience through the integration of governance, lived experience, and knowledge

**DOI:** 10.4102/jamba.v17i1.1988

**Published:** 2025-10-06

**Authors:** Dewald van Niekerk

**Affiliations:** 1 Unit for Environmental Sciences and Management, African Centre for Disaster Studies, Faculty of Natural and Agricultural Sciences, School for Geo- and Spatial Sciences, North-West University, Potchefstroom, South Africa

This edition of *Jàmbá: Journal of Disaster Risk Studies* (Vol. 17 No. 1) features a diverse range of studies from Indonesia and Africa – two regions characterised by both significant risks and remarkable resilience in disaster-prone areas. Located along seismic zones, monsoon regions and transboundary river basins, these areas regularly face hazards such as earthquakes, tsunamis and volcanic eruptions in Indonesia, as well as droughts, floods and urban vulnerabilities in Africa. However, the articles demonstrate that DRR goes beyond hazard monitoring or technical solutions; it is deeply rooted in social, cultural and governance factors. The case studies illustrate how communities, institutions and technologies collaborate to influence preparedness, response and recovery – providing insights that are relevant across borders. Overall, the Indonesian and African experiences reveal common challenges and innovative strategies that contribute to a shared global resilience agenda.

## Integration of sustainability, health and vulnerability reduction

Disasters in Indonesia intersect with chronic vulnerabilities. Akhmad et al. (2025) introduce a sustainability trilogy for drought prevention, linking social, economic and ecological resilience. Similarly, Sakti et al. (2025) show how stunting prevention can be strengthened through risk mitigation programmes, highlighting the inseparability of health, nutrition and DRR. At the urban scale, Mutia et al. (2025) demonstrate how a comprehensive vulnerability index can unpack the multidimensional drivers of pluvial flooding in Langsa. Together, these studies reveal that reducing risk requires systemic integration across sectors.

## Innovation through technology, simulation and artificial intelligence

Technological innovation is a recurring theme in this issue. Ningrum et al. (2025) showcase unmanned aerial vehicle (UAV)-based photogrammetry for tsunami hazard mapping, while Aksa et al. (2025) show that immersive virtual reality (VR) significantly enhances evacuation knowledge and self-efficacy in flood scenarios. Wibowo et al. (2025) extend this horizon further, revealing through a bibliometric review the rapidly expanding role of artificial intelligence (AI) in disaster management, from early detection to social media analysis. These studies emphasise that Indonesia must not only adopt but also adapt cutting-edge tools to local realities, ensuring accessibility and contextual relevance.

## Inclusion of communities, diversity and lived experiences

Effective DRR cannot succeed without recognising social and cultural dimensions. Wijaya et al. (2025) emphasise the role of belief systems and institutional support in shaping preparedness in Merapi’s tourist villages. Dewi et al. (2025) highlight gendered differences in disaster knowledge among students, while Arianti et al. (2025) explore how volunteer helping behaviour provides psychosocial recovery. Saputra et al. (2025) identify multidimensional factors that shape older persons’ resilience to floods in Madura, showing that age, health status and institutional support critically determine outcomes. Solekhah et al. (2025) emphasise how the co-production of knowledge at Merapi and Kelud enhances collective resilience. These contributions highlight the imperative of inclusion, not as rhetoric but as a lived necessity.

## Evolving knowledge and preparedness

Several contributions examine the trajectory of DRR research itself. Ida et al. (2025) document communication gaps during the Mount Semeru eruption, while Adji et al. (2025) examine post-earthquake evacuation failures. At a broader scale, Rohana et al. (2025) employ a bibliometric analysis to trace the evolution of disaster preparedness studies, identifying global trends, research gaps and new directions. This meta-perspective underscores the importance for Indonesia to position its DRR research within global discussions while also drawing on its distinctive experiences.

Across Africa, efforts in DRR and climate resilience are influenced by systemic vulnerabilities and innovative adaptive strategies. The articles in this issue showcase the wide range of African contexts, including drought-prone South Africa, rainfall variability in Namibia, urban hazards driven by climate change in Sierra Leone and the communication issues related to misinformation and social media across the continent. Together, they reveal that Africa’s DRR efforts depend on adaptation, governance and the integration of various knowledge systems.

## Adaptive strategies for drought and climate extremes

Khumalo et al. (2025) examine community-based adaptive mechanisms to drought in KwaZulu-Natal, revealing reliance on borrowing, sharecropping and short-term coping strategies, with limited systemic support. Complementing this, Mpofu, Kgabi and Piketh (2025) analyse rainfall trends in the Cuvelai-Etosha Basin (1968–2018), showing shortened rainy seasons and episodic floods that underscore both uncertainty and urgency in resilience planning. These studies underscore the pivotal role of climate extremes in shaping livelihoods and emphasise the urgent need for anticipatory rather than reactive measures.

## Governance, land-use and urban vulnerability

Urban environments increase the risk levels. Nkosi et al. (2025) examine vulnerabilities in Tswaing informal settlement, Hammanskraal, where illegal electricity hookups and inadequate waste management heighten dangers. Qonono and Lunga (2025) analyse petrol station placement in South Africa’s major cities, revealing widespread violations of safety standards and highlighting the importance of integrating DRR principles into urban land-use planning. Meanwhile, Sam-Mbomah et al. (2025) evaluate disaster risk management (DRM) and climate change adaptation (CCA) in Freetown, Sierra Leone, revealing systemic issues in coordination, communication and enforcement. Collectively, these studies emphasise that African cities face a convergence of governance deficiencies and hazard exposure, which require comprehensive and enforceable policies.

## Communication, misinformation and social media

Effective information flows are essential for preparedness. Cuadra and Cotoron (2025) highlight how misinformation weakens community-based DRM and emphasise the need for digital literacy, local communication networks and trusted leadership. Ejem et al. (2025) extend this analysis to a continental level, investigating how social media can be integrated into African climate disaster management through a force-field analysis of enabling and limiting factors. These findings reveal a paradox: while communication technologies have the potential to improve disaster response, a lack of trust and capacity can turn them into factors that increase vulnerability.

## Indigenous knowledge and inclusive approaches

Motsumi and Nemakonde (2025) underscore the predictive importance of indigenous early warning signs in South Africa’s Northern Cape, such as lunar cycles and bird behaviour, as valuable complements to meteorological forecasts. Their research aligns with global efforts to integrate scientific and indigenous knowledge, emphasising that resilience encompasses cultural and social dimensions, not just technical ones. Concurrently, Mahembe and Mutezo (2025) highlight the necessity for customised operational risk management frameworks tailored to small tourism businesses, illustrating that disaster preparedness must be sector-specific and adjusted according to resource levels.

The insights from Indonesia and Africa, despite their different locations and hazard profiles, highlight a common theme: resilience develops through the combination of knowledge, governance and lived experience. Whether by incorporating sustainability into health and development plans, leveraging technology and communication tools creatively or engaging diverse communities and knowledge systems, the approaches to reducing risk are both local and global. This edition emphasises that DRR depends not just on technical measures but also on trust, participation, and coordinated action across different scales and borders. Learning from these experiences reminds us that creating safer futures involves not only understanding hazards but also strengthening social cohesion and institutional structures that enable societies to adapt, recover and prosper.
